# A rare case of narrow QRS complex tachycardia

**DOI:** 10.1007/s12471-014-0615-z

**Published:** 2014-11-12

**Authors:** L. E. Swart, Y. S. Tuininga

**Affiliations:** Department of Cardiology, Deventer Hospital, Nico Bolkesteinlaan 75, 7416 SE Deventer, the Netherlands

**Fig. 1:**
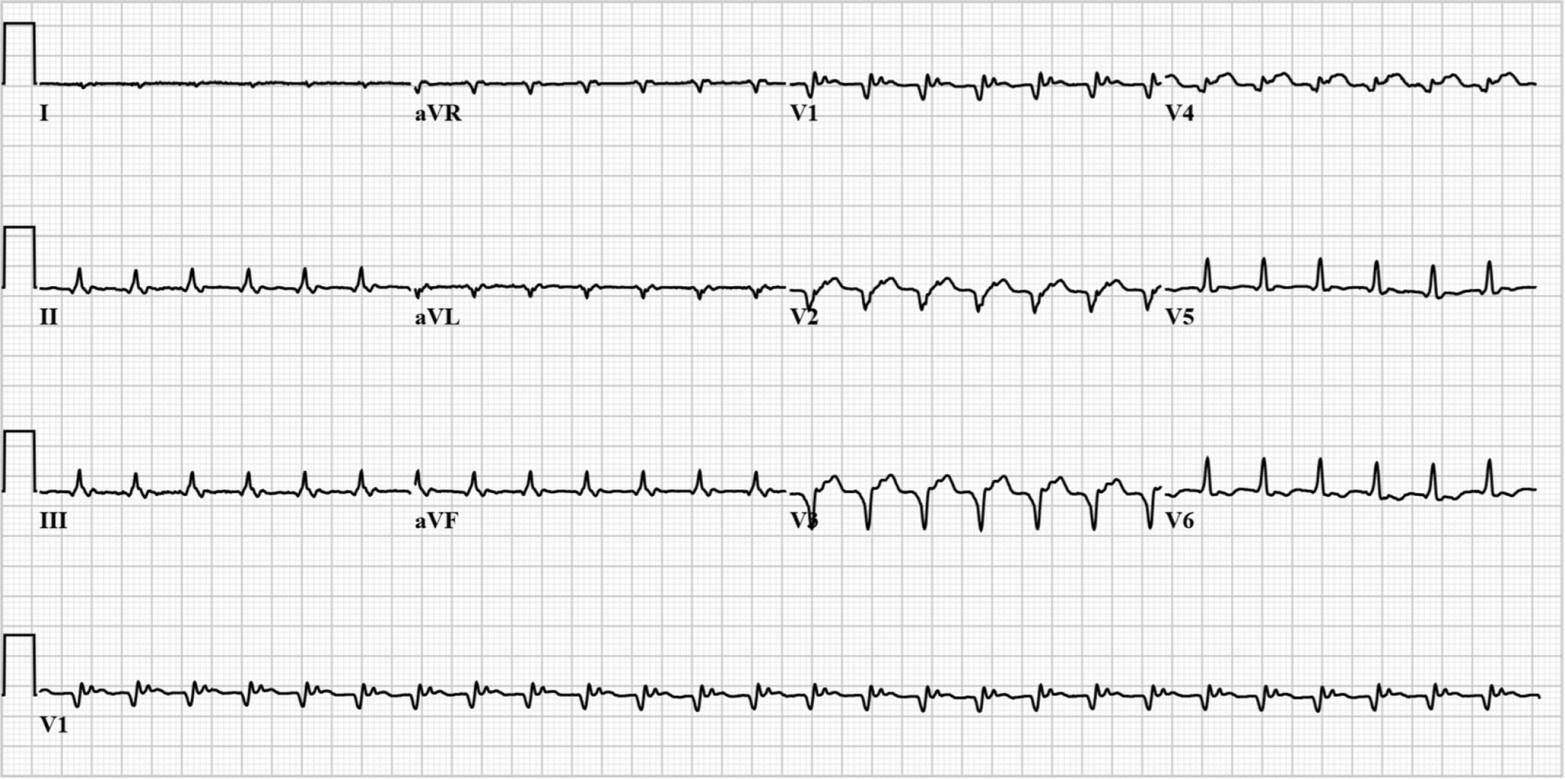
First standard 12-lead ECG at presentation. Ventricular rate: 160 bpm, QRS duration (calculated): 106 ms

We present the case of a 63-year-old female patient who, 2 months earlier, had been diagnosed with a severe ischaemic cardiomyopathy (left ventricular ejection fraction of 18 % on cardiac MRI) due to a large semi-recent transmural left anterior descending artery infarction. She was referred to our coronary care unit because her physical condition had been declining rapidly over the previous 2 days, with her main complaint being dyspnoea on the slightest physical exertion (NYHA III). She experienced no dyspnoea at rest, nor orthopnoea, chest pain or palpitations. On admission she had a regular pulse of just over 150 beats/min, a blood pressure of 100/60 mmHg, an SpO2 of 100 % and there were no physical signs of congestive heart failure. The ECG at presentation is shown in Fig. 1. What is your most likely diagnosis?

**Fig. 2:**
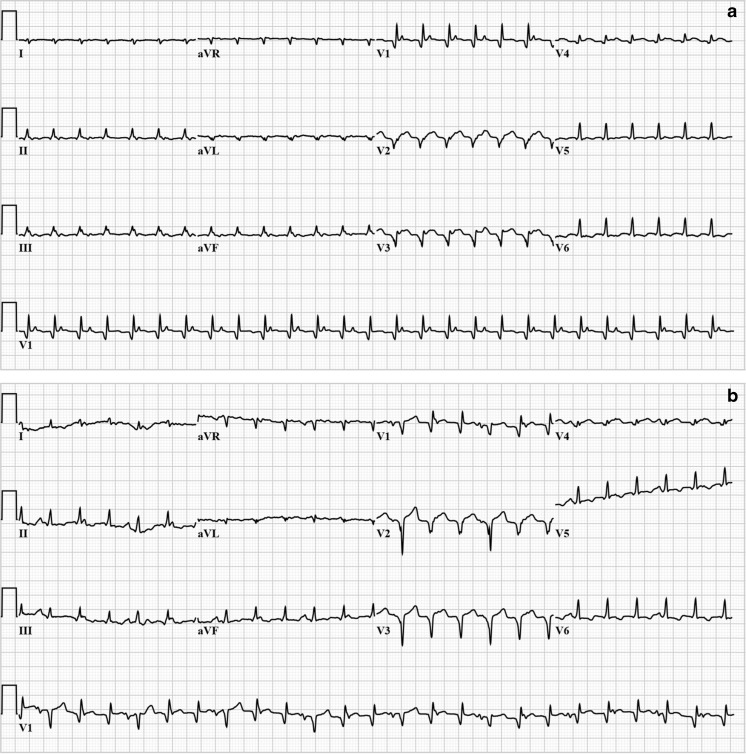
**a** Second standard 12-lead ECG, a few minutes after adenosine infusion, and the **b** third standard 12-lead ECG, shortly thereafter

Intravenous adenosine bolus of up to 18 mg did not have any effect on the rhythm and her vital signs remained unchanged. A few minutes later, and 5 min thereafter, a second and third ECG were obtained (Fig. 2). Do these change your diagnosis?

You will find the answer elsewhere in this issue.

